# Utility, Value, and Benefits of Contemporary Personal Health Records: Integrative Review and Conceptual Synthesis

**DOI:** 10.2196/26877

**Published:** 2021-04-29

**Authors:** Umar Ruhi, Ritesh Chugh

**Affiliations:** 1 Telfer School of Management University of Ottawa Ottawa, ON Canada; 2 School of Engineering & Technology Central Queensland University Melbourne Australia

**Keywords:** electronic personal health records, PHR, functionality synopsis, value analysis, consumer health informatics

## Abstract

**Background:**

Contemporary personal health record (PHR) technologies offer a useful platform for individuals to maintain a lifelong record of personally reported and clinically sourced data from various points of medical care.

**Objective:**

This paper presents an integrative review and synthesis of the extant literature on PHRs. This review draws upon multiple lenses of analysis and deliberates value perspectives of PHRs at the product, consumer, and industry levels.

**Methods:**

Academic databases were searched using multiple keywords related to PHRs for the years 2001-2020. Three research questions were formulated and used as selection criteria in our review of the extant literature relevant to our study.

**Results:**

We offer a high-level functional utility model of PHR features and functions. We also conceptualize a consumer value framework of PHRs, highlighting the applications of these technologies across various health care delivery activities. Finally, we provide a summary of the benefits of PHRs for various health care constituents, including consumers, providers, payors, and public health agencies.

**Conclusions:**

PHR products offer a myriad of content-, connectivity-, and collaboration-based features and functions for their users. Although consumers benefit from the tools provided by PHR technologies, their overall value extends across the constituents of the health care delivery chain. Despite advances in technology, our literature review identifies a shortfall in the research addressing consumer value enabled by PHR tools. In addition to scholars and researchers, our literature review and proposed framework may be especially helpful for value analysis committees in the health care sector that are commissioned for the appraisal of innovative health information technologies such as PHRs.

## Introduction

Among the many technology applications available to individuals today for managing their own health and wellness, electronic personal health records (PHRs) offer a valuable means to facilitate active participation of health care consumers, including patients and their caregivers. By virtue of their potential capabilities to help individuals track their health conditions, provide access to patient medical record information (PMRI), and offer communication tools to interact with health care providers, PHRs have been regarded as a paradigm shift toward consumer-centric and patient-oriented health and medical services [1,2].

Although the consumer adoption of PHR systems has been slower than originally expected [3,4], these technologies are gaining traction in many countries worldwide [5-9]. Government eHealth initiatives in many countries are currently focused on the implementation of these technologies to foster greater patient engagement with personal health information (PHI) management and care coordination. An example of such an initiative is the Stage 3 Meaningful Use program under the US Health Information Technology for Economic and Clinical Health initiative. This program calls for improving patient engagement through functionality, such as patient access to medical records, patient communication tools, and interoperability with hospital electronic health records (EHRs) [10,11]. Across the border, in Canada, several federally funded projects sponsored by the Canada Health Infoway are also geared toward the deployment of consumer-focused digital health technologies, including patient health information records, patient-physician communication tools, and remote patient monitoring [12]. Along similar lines, the p-medicine and eHealthMonitor projects funded by the European Union also aim to support personalized medicine through technologies such as PHRs [7].

The overarching vision behind PHR technology offerings is to enable patient empowerment, reduce health care costs, and provide better continuity of care [3] through access to timely, reliable, and comprehensible health information for patients and streamlined communication between patients and health care providers [13,14]. The objective of this paper is to offer a review of the utility, value, and benefits of PHR systems through a discussion of their features and functions and to deliberate how PHR functionality can potentially translate into value for the health care consumer and benefits for the health care system as a whole. Our review comprises both an analysis and a synthesis-oriented exposition on the current landscape of PHR technologies. The *Methods* section outlines our review approach and the ensuing structure of this study.

## Methods

### Overview

In characterizing the type of review offered in this paper, our discussion aligns with an integrative review, in which literature pertinent to a subject area is critically analyzed and synthesized to theorize alternative perspectives of the subject [15]. Although integrative literature reviews may serve multiple purposes, their essence is to review existing literature to elicit new insights, inquiries, or answers through the integration and synthesis of existing literature [16]. Integrative reviews are commonly used in health research [17-19], and such reviews have been purported to enhance the development of health care theory, policy, and practice [19]. Our integrative review was structured along the following 5 recommended phases [19]: problem identification, literature search, data evaluation, data analysis, and presentation of the results. The procedures followed are summarized below.

### Problem Identification

Specific to our study, the purpose of our review is to extensively research pertinent PHR literature and provide an assessment of the product utility, consumer value, and industry benefits of these systems. Toward this, and in line with integrative reviews, we developed a protocol for the search and selection of relevant literature [15,16], deliberated the capabilities of PHR systems using several lenses of analysis, and then classified and synthesized a typology to formulate conceptual frameworks that explicate the utility and value of PHR systems. Typologies that offer a conceptual classification of constructs are recommended as useful theory-building tools [20] and a valuable form of synthesis in integrative reviews [15].

To guide our review process, we formulated 3 research questions that we aimed to answer through our analysis and synthesis of the extant literature. Table 1 below is the three-step approach that we adapted for our review based on suggested guidelines for the reviews of emerging HITs [21,22]. The *Results* section of this paper discusses the findings and outcomes from our review, as noted in Table 1.

**Table 1:** Research questions and guidelines followed for the review and synthesis.

Review perspective	RQ^a^	HIT^b^ assessment review guidelines	Review and synthesis outcomes
Product utility	RQ1. What features and functions are available in contemporary PHRs^c^, and how has this functionality evolved over time?	1. Technology definition and literature search	Literature selection procedure PHR working definition Functional utility model of PHR technologies
Consumer value	RQ2. What is the potential value of various PHR functionalities to health care consumers?	2. Conceptual analysis and framework formulation	Functional utility model of PHR technologies PHR consumer value framework
Industry benefits	RQ3. How can the mainstream deployment and use of PHR systems translate into benefits for the health care system as a whole?	3. Reflective synthesis and summary	Value propositions and benefits of PHR systems

^a^RQ: research question.

^b^HIT: health information technology.

^c^PHR: personal health record.

### Search Strategy

Our academic article search was conducted using digital library databases, including PubMed, Web of Science, ScienceDirect, and Scopus. In addition, to ensure the breadth and validity of our search results, we explored the publications cited in previous scoping and systematic reviews of PHRs [23-25] and included any relevant articles that had been overlooked in our own search.

Our search techniques used various terms and keywords related to PHRs, including acronyms as well as expanded terms, such as PHR, personal health record, EPHR (electronic personal health record), patient portal, personal medical record, personally controlled health record, PCHR, personal health information, and PHI.

### Data Evaluation

Both authors independently screened titles, keywords, and abstracts to determine whether publications should be included in the review. Our review included studies that explicitly discussed features, functions, utility, value, and benefits of electronic PHRs, whereas it excluded publications focusing on paper-based PHRs or studies solely focusing on psychosocial aspects of end users’ PHR adoption or technical system design practices for PHRs. Following the first round of screening, we refined our search criteria and examined articles pertaining to consumer health informatics as a general field of study. Our initial review indicated that some publications pertaining to consumer health informatics directly discuss the benefits of PHR technologies [13,26-30]. In the second round of screening, each author assessed mutually exclusive but collectively exhaustive subsets of all publications identified as potentially relevant, and we ensured that the articles were indeed pertinent to our review.

### Data Analysis and Synthesis

Following the selection of relevant literature, our review process began with an iterative concept-centric analysis of the attributes and benefits of PHR systems. We analyzed the literature at the product level by identifying various features and functions of PHR systems described in the extant literature, at the consumer level by deliberating the value of various PHR system functionalities, and at the industry level by identifying the benefits provided by PHR technologies to various health care industry constituents. A codebook was created to facilitate the analysis and extraction of data into systematic categories. The authors collaborated on the conceptual synthesis of the 3 classification systems for functional utility, consumer value, and industry benefits. These conceptual classifications were refined iteratively through simplification, abstraction, and focusing procedures, constituting the constant comparison method commonly recommended for integrative reviews [19].

### Presentation and Paper Structure

In the final phase of the integrative review, the results from our analysis and synthesis were summarized and depicted using visual models and concept matrices. These are presented and discussed in the *Results* section of this paper.

We first provide a working definition of PHRs that was used as a touchstone to guide our literature search and subsequent discussion. Drawing upon that definition, we retrieved relevant peer-reviewed publications and industry reports that discussed the functionality, utility, value, and benefits of PHR technologies.

The outcome of our review of PHRs from a product utility perspective comprised an evaluation of various features and functions of PHR systems. The output from this evaluation is conceptualized as a high-level functional model of PHRs that summarizes the myriad of features and functions available in contemporary PHR systems.

Next, we discuss the capabilities of PHR systems from a consumer value orientation by juxtaposing the functionality of PHR systems alongside health care delivery activities ranging from prevention to the diagnosis and ongoing management of illnesses.

Finally, the *Results* section provides an industry-level viewpoint that summarizes various value propositions and benefits related to the use of PHR technologies at the micro, meso, and macro levels. The synthesis offers a discussion of how the effective deployment and use of PHR technologies can potentially translate into benefits for different constituents in the health care delivery chain, including consumers, providers, payors, and public health agencies.

## Results

### Defining Characteristics of PHRs

As a working definition, this paper adopts one of the earliest and most commonly cited characterization of a PHR as “an electronic application through which individuals can access, manage and share their health information and that of others for whom they are authorized, in a private, secure and confidential environment” [31]. In addition, PHR are sometimes referred to as personally controlled health records (PCHRs) comprising information and communication technologies that can potentially help all types of end users in maintaining health and wellness and specifically facilitate patients in managing their ongoing illnesses [32].

To further delineate the representative attributes of PHRs, we also differentiate between 3 similar yet distinct technologies related to patient records: electronic medical records (EMRs), EHRs, and PHRs. Depending on the health care setting, although these 3 technologies may be used as components in an integrated health information system (HIS), each of them can be differentiated from the other based on its custodianship and level of patient centricity. EMRs are often considered as digital versions of paper charts in a clinician’s office [33]. These patient medical records in this instance are provider-centric [33,34] and are rarely accessible to other health care providers or to the patients themselves. In contrast, EHR systems offer a broader view of a patient’s care by facilitating integration with HIS beyond the organization that originally collected and compiled the patient information [33]. These systems can aggregate patient data from multiple health care facilities to create a unified patient record that can be accessed by health care providers [35,36]. Finally, PHRs function under the custodianship of patients or their caregivers, and these systems comprise full or partial health information about patients over their lifetime [35,37]. Hence, PHRs specifically pertain to digitally stored health care information about an individual patient under the control of that patient or their caregiver [7,38], whereas EMRs and EHRs are typically maintained by health care providers or payor organizations [39].

Drawing upon these characteristics of PHR systems, this study adopts a consumer-oriented perspective and uses the term PHR to refer to both the underlying patient record and its data elements as well as the software that provides functionality to maintain that record. As such, we do not differentiate between the data (PHR) and PHR-S (software components of PHR), as sometimes done in the industry standard documentation such as HL7 (Health Level Seven) [40,41]. Furthermore, although we note that there may be differences among PHR systems in terms of front-end technology features, back-end information sources, patients’ scope of access, and storage locations of online records, we consider electronic access (desktop, web, or mobile) and patient control over health records to be the defining characteristics of PHR systems.

To help understand the functional scope of the current PHR systems, researchers have classified these technologies into 3 main categories: *standalone*, *tethered*, and *interconnected* [32,37]. The main attributes that differentiate these categories are data control, record portability, and system interoperability. The key differences among these categories are outlined below.

*Standalone* PHRs require users to manually enter data to populate their own health information and medical history. Hence, the content of these applications is under direct physical control and ownership of the consumer. These PHR systems require a considerable long-term commitment from end users who need to be motivated to maintain their PHI in an accurate and complete fashion [37,42]. Although these technologies may be portable in allowing users to access their PHI anytime and anywhere, they lack interoperability because data must be manually imported or exported from other HIS.

*Tethered* PHR systems are typically offered as extensions of a health care institution’s own back-end EHR or EMR system, providing users access to parts of their own EHRs. These systems are also referred to as patient portals. In addition to providing access to patient data, these systems may also include additional functionality, such as communication tools for email, messaging, appointment scheduling, and prescription renewals [37]. Access to these PHRs is typically provided through a web portal interface [32,43]. The data in tethered PHR systems are under the control of the health care provider, hence limiting the portability of patient records, and these technologies may not be fully interoperable with other HIS.

*Interconnected* PHRs are often described as the ideal or preferred type of PHR in terms of data control, record portability, and system interoperability [7,37,44,45]. These systems can usually be populated with patient information from a variety of sources, including physician EMRs, hospital EHRs, insurance carriers, health plan sponsors, labs, and pharmacies [29,37]. In addition, users can enter their own information in the selected areas of the PHR. These PHR systems offer consumers adequate control over parts of their health records and also alleviate the need for manual data entry. In addition, because of the established electronic linkages among some HIS, records can usually be easily transferred from one provider to another. Despite the limited offerings in this space, the functionality of these integrated systems is expected to translate into a wider range of convenience benefits and improved health outcomes for consumers as well as operational efficiencies for providers.

### Product Utility of PHR Systems

On the basis of the discussion above on different types of PHR systems, one may be led to believe that tethered or interconnected PHRs offer considerably advanced functionality in comparison with standalone PHR systems. Although this is certainly true in the context of system capabilities that require back-end integration with provider EMR or EHR systems, there may be a range of other PHR features and functions that do not depend on such integration, and these can be offered through stand-alone PHRs just as well. For example, certain stand-alone PHR products provide a deeper functionality related to health resource libraries, patient-centered health monitoring, and linkages with web-based support groups—features that do not necessarily need high levels of system interoperability with other HIS.

**Figure 1:**
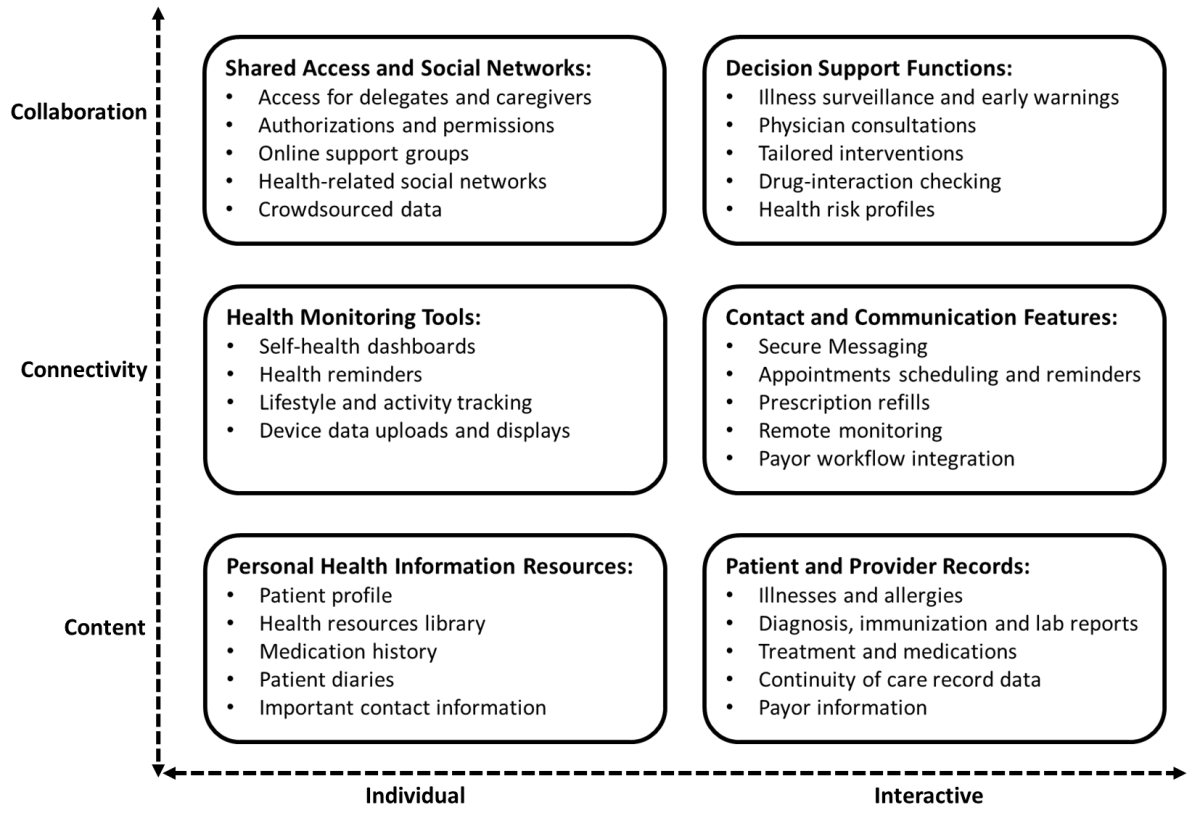
A high-level functional utility model of personal health record systems.

In this section, we draw upon the extant academic literature as well as the current industry PHR software offerings to provide an overview of various functionalities that may be available in contemporary PHR systems. To aid our discussion, we organize the different PHR capabilities into different categories based on the consumers’ modalities of use and functional characteristics of system features and functions. Figure 1 depicts the conceptualization of a high-level functional utility model of PHR systems. The elements of the model are briefly described below. It should be noted that our model does not aim to provide a system specification or technical architecture of PHR systems. These have been described elsewhere in the extant literature [30,45,46]. Our model simply aims to provide a scaffold to aid a high-level understanding of PHR systems at the consumer level.

In terms of modalities of use, our model differentiates between the *individual* and *interactive* modes of using PHR functions. Although individual modes of use entail highly personalized user-initiated tasks, interactive modes usually comprise bidirectional exchanges between the consumer and other care delivery constituents, including physicians, providers, or payors. Most of the individual tasks are performed in an asynchronous style with frequent individual interventions, whereas interactive exchanges usually occur in a dynamic and synchronous fashion.

Our model also characterizes PHR functions as being primarily *content*-, *connectivity*-, or *collaboration-oriented*. Content-rich features refer to PHR technologies that are primarily used for information management. Information can be pushed automatically or retrieved on an as-needed basis or it can be maintained within the PHR by users on their own. In connectivity-based applications, information is exchanged, and transactions are conducted in a 2-way flow between applications, devices, organizations, or people. Finally, collaboration-based mechanisms subsume other functional modes and offer tools for interpersonal exchanges and decision support, thereby enabling consumers to proactively manage their health and wellness. On the basis of these criteria for classification, we categorized various PHR features and functions into 6 groups: PHI resources, patient and provider records, health monitoring tools, contact and communication features, shared access and social networks, and decision support functions.

#### PHI Resources

At their core, most PHR systems comprise a repository of PHI that allows consumers to maintain their own profiles and medical history data. Various tools such as digital diaries to manage the lists of drugs and track personal data such as weight, glucose, and cholesterol levels allow consumers to exercise control over their medical information [47-49]. Additional functionality with links to web-based health information can help consumers create a library of health information resources pertinent to them [50,51].

#### Patient and Provider Records

For PHRs with electronic links to other HIS from providers, pertinent PMRI can be seamlessly added to the PHR system. Data from patient diagnosis, treatments, and medications can be added from physicians’ EMRs or providers’ EHR systems [50,52-57]. Patient health summary standards such as the CCR (continuity of care record) can provide guidelines for PHR pertinent data that would provide a holistic view of patient care and consequently improve the portability of patient health information [42,55,58].

#### Health Monitoring Tools

Beyond self-managed health information, many PHR technologies also facilitate connectivity with a range of medical and lifestyle tracking devices. Data from these devices can be uploaded to PHRs to enable consumers to keep track of their health and wellness [55,56,59-62]. In addition, these behavior management tools can help consumers track their health indicators via a dashboard style interface and also set up various notifications and alerts for any anomalies or items that require their attention [63-65].

#### Contact and Communication Features

The tools in this category are considered extremely useful by consumers for interconnected PHRs linked to provider EMR and EHR systems [7,45,52,59,60,64]. Although features such as patient-physician and patient-provider secure messaging and appointment scheduling provide convenience to users, other tools for prescription refills and insurance claims processing can help streamline process workflows for all constituents in health care delivery [66-68]. Advanced PHR offerings also provide telehealth features for patients to provide the results of basic health assessments from home and to transfer data from connected medical devices [37,64,69].

#### Shared Access and Social Networks

Most PHR systems provide a core set of collaboration tools to help consumers share their health information with other authorized people, including caregivers and designated family members. They do so by delegating access rights and permissions to the specific parts of their PHR [37,64,69]. More recently, social networking tools have been integrated with some PHRs to provide patients with more access to information from practitioners as well as from other patients with similar medical conditions [29,70]. The range of possibilities for social networking features in PHR offerings span a wide spectrum from basic moderated health discussion forums for questions and answers [59] to sophisticated sites that crowdsource patient data from connected devices to foster an active dialog among patients or to contribute to further research about illnesses [71].

#### Decision Support Functions

In interconnected PHR systems, collaborative interactions between patients and clinicians can be enabled through decision support features that include illness surveillance, virtual consultations, and computerized tailored interventions [29,72]. In addition, rule-based engines can also provide input to the decision-making processes through tools such as patient health risk profiles and drug-interaction checking [7,73-75]. By using patient data from other parts of the PHR and leveraging practitioner expertise, such tools can help in evaluating the harms and benefits of specific treatment options [37,63,76], issue health warnings through personalized clinical decision support (CDS) notifications [29,77], and recommend alternative treatments [37,73,78]. Recent studies have also shown that personalization-focused PHR functions such as tailored interventions with highly individualized communication, therapy, or medications can be extremely effective in inducing behavioral changes and improving patient health [79,80].

### Consumer Value of PHR Systems

Drawing upon the review of the features and functions of PHR systems at the product level, this section discusses a consumer-centric viewpoint of the potential value that might be realized through the effective use of these technologies. To facilitate this viewpoint, we appropriate the conceptual framework of the *care delivery value chain* (CDVC) [81-83], which offers a systematic approach to delineate and analyze health care services and activities that jointly determine the overall success of health outcomes for consumers [81]. According to the CDVC framework, the value for the health care consumer is determined by the results and outcomes rather than the inputs and volume of the range of health care services provided [84]. Using a similar reasoning, we maintain that in the case of PHRs, the consumer value is determined not by the functions and features of technologies per se but also by their potential to address the needs, expectations, and preferences of consumers during the various health care delivery activities. The CDVC classifies these activities into 6 main areas: prevention, diagnosis, preparation, interventions, recovery, and ongoing management of illnesses [82,83].

**Figure 2:**
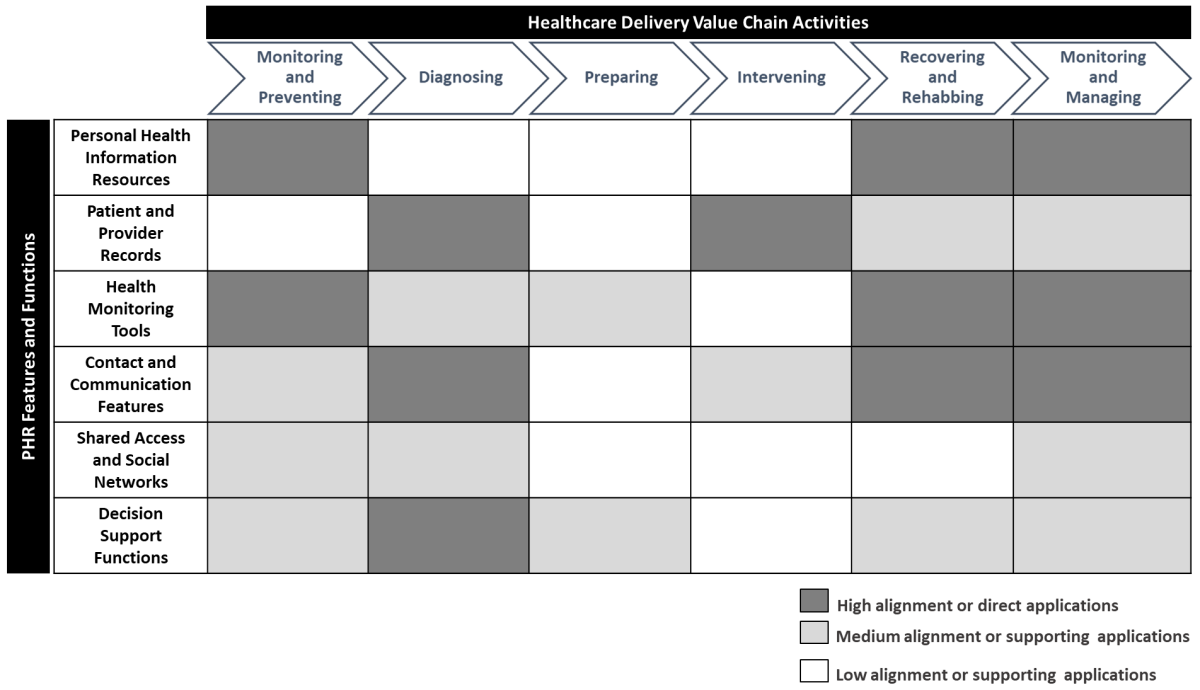
Personal health record consumer value: a mapping of personal health record functionality to the health care delivery value chain. PHR: personal health record.

Figure 2 presents a visual depiction of our conceptualization of the PHR consumer value framework that juxtaposes various PHR features and functions (described in the *Decision Support Functions* section) alongside the CDVC-defined 6 core activities in the health care delivery value chain. The relationships between PHR functionality and health care activities are depicted as lightly or darkly shaded intersections. The latter represents a high (direct) alignment or mapping between specific PHR functionality and health care delivery activity, as evidenced by strong support in the extant literature. To determine the strength of mapping between PHR functional categories and CDVC activities, we identified different use cases for PHR features and functions from the extant literature. These use cases are listed in Multimedia Appendix 1, which serves as a foundation for the summary depicted in Figure 2. A brief overview of the applications of PHR functionalities in different health care delivery activities is provided below.

#### Monitoring and Preventing

These activities are primarily concerned with tracking an individual’s current conditions and assessing health risks to proactively prevent or reduce the seriousness of illness or injury [81]. Comprehensive patient profile and medical history information sourced from personally reported data as well as patient clinical records can directly help in the early detection of illnesses or reducing the need for medical treatments [7,48]. Furthermore, the integrated use of health devices and self-management dashboards can help track lifestyle-or illness-related risk factors such as diet patterns, blood pressure, and glucose levels, which can help support preventive health care activities [48,55,59,85,86]. Social networks can also help support the proactive posture of patients by helping source relevant information about health conditions and treatments from practitioners and other patients [29,71]. Decision support tools offer an additional functionality to help with the proactive detection and resolution of potentially threatening health conditions through CDS advisories and virtual counseling from experts about potential future health risks [55,74,75,81].

#### Diagnosing

Diagnosing activities in the care cycle comprise a range of processes, such as laboratory testing, medical history evaluation, consultations with specialists, and the formulation of treatment plans. As depicted in Figure 2, there is a strong support in the extant literature that multiple PHR functionality groups have direct or indirect applications in diagnosis-related care activities. The ready availability of PMRI in interconnected PHR systems can help facilitate these activities [47,52,53,70]. These activities can also be enabled through decision support tools, such as virtual physician consultations and computerized tailored interventions [29,55,70,75]. Finally, in terms of supporting functionalities, personally tracked and self-reported personal information and family medical history shared through the PHR can also help in improving the overall quality of diagnostic processes [47,55,70,75,87].

#### Preparing

These activities refer to all setup procedures and processes that need to be completed before medical intervention. In the original CDVC framework, the authors note that this set of activities is often overlooked in the health care system [81,82]. Perhaps this is why our review also yielded very little direct evidence of PHR applications in this area. Nonetheless, we believe that PHR functionality related to health monitoring tools and decision support functions can help in supporting such preparatory activities. The former can help with the tracking of important health data from lifestyle health data dashboards and connected medical devices [61,62,65], whereas the latter can facilitate reviews of health profiles, interactions with specialists, and verification of potential drug interactions [29,73].

#### Intervening

Intervention processes and procedures are targeted at reversing or mitigating a health condition [81], and they typically include the initiation of therapy, treatment, or medication and the management of potential infections or associated illnesses. PHRs can play an important role in improving the quality of medical interventions by providing up-to-date medical history and PMRI across various points of care and by facilitating continuity of care [42,54,66,68]. In addition, contact and communication features in PHR systems can help patients receive regular and situational counseling on treatment and prognosis and can help attending physicians ensure treatment compliance [81,83].

#### Recovering and Rehabbing

These services are an essential component of care for all medical conditions [81], and the procedures are important for ensuring effective recovery and positive health outcomes for patients in the long term. PHR systems can help in achieving these health outcomes through an improved health monitoring of risk factors and lifestyle data [55-57,61,65,86,88,89] and by seamlessly connecting patients to providers for referrals, follow-ups, and prescription refills [63]. These features are also complemented well by decision support functions that can facilitate the development of tailored interventions and help in tracking and resolving treatment side effects [62,73,75,90].

#### Monitoring and Managing

Activities that constitute the final part of the care delivery chain aim to manage patient conditions and monitor therapy compliance on an ongoing basis [83]. As shown in Figure 2, PHI resources, health monitoring tools, and contact and communication features can potentially play a significant role in enabling these monitoring and managing activities [56,57,85,89,91,92]. Multiple studies pertaining to chronically ill patients using PHR technologies have demonstrated the usefulness of features such as personal logs and links to educational resources as well as tools such as web-based appointment scheduling, meeting reminders, and email communication with health care professionals [85,91,93]. Together, these tools can play a vital role in minimizing long-term health risks related to chronic illnesses [56,57,82,89,92].

Overall, the effective deployment and adoption of PHRs can potentially enable improved integration across health care activities that constitute the full cycle of care for a consumer. Such integration across the entire chain has been posited as the major driver of health care consumer value [83].

Our analytical framework highlights the similarities and complementarities among various PHR functions and features by conceptualizing the direct and supporting applications of PHRs in different health care activities. Furthermore, a circumspect inspection of the framework shows that the consumer value from contemporary PHRs primarily relates to care activities with a proactive health management orientation. Monitoring activities at the beginning and end of the care delivery chain has a high number of associated PHR applications, followed by activities related to diagnosis and recovery. From the viewpoint of various PHR functional categories, contact and communication features appear to have the most recurrent use cases, followed by personally and clinically sourced health record information as well as health monitoring tools. Our analysis supports previous research that indicates that PHR users consider connectivity features that facilitate health care processes to be the most useful tools [48,94].

### Industry Benefits of PHR Systems

Having discussed the functionality of PHR systems and their applications in various health care activities, this section of the paper offers a summary of the benefits of PHR systems for the health care industry. In deliberating these benefits, we underscore our assertion that the value-producing potential of PHRs is not only dependent on the adoption of these technologies by consumers but also on the active participation for the provision and use of these technologies by multiple health care delivery constituents, such as hospitals, labs, pharmacies, insurance companies, and government agencies. Consequently, the effective deployment and adoption of PHRs can result in a variety of benefits for these constituents [95].

The lens of analysis used to outline the industry benefits of PHRs is based on an extension of the health care value chain described in the *Monitoring and Managing* section. However, rather than focusing on care delivery activities, we adopt a channel partner perspective and highlight the benefits of PHR technologies to various entities that comprise the health care system. Using standard health care industry nomenclature [96,97], we refer to providers as any clinicians, allied health professionals, or organizations that render direct health care services to consumers; payors as entities that finance the cost of health services (eg, insurance carriers or health plan sponsors); and public health agencies as government institutions concerned with research and policy issues for the social well-being of communities as a whole [97].

Table 2 offers a review summary of the industry benefits of PHRs by outlining the core value propositions and the principal benefits of these technologies to different constituents. Value propositions pertain to micro-level benefits for consumers, meso-level benefits for providers and payors, and macro-level benefits in the realm of public health. These are briefly discussed herewith.

**Table 2:** Value propositions and benefits of personal health record systems to health care delivery constituents.

Value propositions and benefits			Literature support		Health care delivery constituents						
					Consumers		Providers		Payors		Public health agencies
**Consumer empowerment and patient engagement**											
	Promote consumer health education	[14,50,57,68,76]		✓^a^		✓				✓	
	Enable patients to become informed health care consumers	[5,42,44,66,98]		✓						✓	
	Enhance understanding of medical conditions	[29,42,57,66,74,75,99,100]		✓		✓					
	Simplify and clarify patient instructions	[29,42,57,66,74,75,99,100]		✓		✓					
	Provide a greater control over health outcomes	[3,56,101,102]		✓							
	Offer convenient self-health management	[21,56,68,86,89,103,104]		✓							
	Facilitate self-efficacy via cues for patient action	[13,102]		✓							
**Health care communication**											
	Improve patient-physician or provider communication	[14,42,105]		✓		✓					
	Timely information sharing for clinical decisions	[68,85,90,103]		✓		✓					
	End-to-end care delivery involving multiple constituents	[44,55,68,89,106,107]		✓		✓		✓		✓	
**Process efficiencies and cost effectiveness**											
	Increased portability of patient records	[44,57,63,68,89,108,109]		✓		✓		✓			
	Reduced cost of chronic disease management	[32,110]		✓		✓		✓			
	Greater medical information validity and accuracy	[42,59,75,76,111,112]		✓		✓		✓		✓	
	Save patient, physician, and provider time	[14,32,57,85]		✓		✓		✓			
	Reduced cost of duplication of tests and procedures	[42,59,113]		✓				✓		✓	
**Enhanced quality of care**											
	Increased patient safety considerations	[90,103,114]		✓		✓				✓	
	Improved handling of emergency situations	[115,116]		✓		✓					
	Extended durability of patient data	[32,104,109]		✓		✓				✓	
	Early identification of patient risks and health susceptibilities	[76,115,117,118]		✓		✓		✓			
**Public health outcomes**											
	Reduced burden on health care system and resources	[3,55,59,63]				✓		✓		✓	
	Enhanced care for underserved communities and populations	[3,59,63,95]		✓		✓				✓	
	Facilitate care in public health emergencies	[119,120]		✓		✓				✓	
	Support public health research	[71,90,121,122]								✓	
	New avenues for epidemiology surveillance and screenings	[71,90,121,122]								✓	

^a^Mapping of value propositions and benefits of personal health records to various health care delivery constituents.

#### Consumers

From the perspective of *consumers*, PHR users are not only likely to be better informed about their health conditions [5,63,123], but they also actively participate and increasingly contribute toward their own health management activities [57,102]. PHRs have the potential to provide patients with a better understanding of health information and clearer health care instructions [74,100]. PHRs can also assist users in monitoring daily self-care activities and enable patients to collaborate and share their experiences with their providers and caregivers. As an integrated patient-centered technology, PHRs also offer the means for increased patient engagement through improved provider-patient and physician-patient communication [14,54,66,68,74], thus leading to greater personalization of care [3].

#### Providers

From the perspective of *providers*, a primary benefit of PHR systems is that these technologies address a significant gap in the current health information exchange mechanisms. In the absence of stable and formal technology standards that allow the transfer of patient records from one provider to another, PHRs can offer an alternative means to achieve this purpose [44,63,107]. Patients can access their health records as, when, and where needed. PHR systems can also help reduce health care costs and inefficiencies, especially those associated with inaccurate information and effort duplication [42,57,59,113]. Patients can directly verify health data, and a complete access to patient history from across providers can assist in avoiding unnecessary laboratory tests and medical procedures.

#### Payors

*Providers* and *payors* can also benefit from the patient adoption of PHRs because the use of these systems is likely to improve patient safety through an early identification of health risks [76,90,103,114], reduce the cost of chronic disease management [32,110], and enable health care institutions to better handle emergency situations [115,116]. Access to unified PMRI across health care providers can help alleviate medical treatment disruptions for patients with chronic conditions [124], and features such as remote monitoring and eHealth consultations can enable earlier and efficient hospital discharge and long-term patient monitoring processes [64,69].

#### Public Health Agencies

From the viewpoint of *public health agencies* at a macro level, the mainstream adoption of PHRs can lead to a variety of benefits for population health [95]. These technologies can help in reducing health care disparities across demographic, economic, and regional divides, and these technologies offer a means of access to high-quality health care for all [124]. By helping overcome structural barriers to quality health care, PHR technologies can potentially improve the health status of underserved communities and populations [69,124]. From a cost perspective, proactive care delivery made possible through these technologies can help in reducing the burden on public health institutions and resources [3,59,63,76].

Finally, from a population health research standpoint, consumer consent to sharing health care information and the subsequent widescale accumulation of PHR data have the potential to act as a valuable source of public health information for promoting healthy lifestyles and for detecting and preventing infectious diseases [119]. Through appropriate privacy and consent mechanisms, patient data available through an integrated health care network can be used to facilitate public health research on individuals and their communities as well as help with regional and global illness surveillance and screenings [55,90,121].

Overall, PHR systems can play a transformative role in facilitating complex information management processes across various health care delivery constituents. The mainstream deployment and adoption of these technologies has the potential to improve clinical and population health outcomes by streamlining medical and operational processes across the health care system.

## Discussion

Although several previous studies on PHR technologies have alluded to a distinction among the functionality, utility, and value of these technologies [5,47,125], our review of the literature did not reveal any formal treatise of this subject at a theoretical or an empirical level. Our study aims to address this gap through a review and synthesis of the literature.

This paper presents a review of the extant literature on PHR systems, with the objective of providing an overall assessment of the functionality, utility, value, and benefits of contemporary PHRs. Toward this end, we offer a conceptual high-level functional utility model of PHRs outlining their features and functions categorized according to different use modalities. In addition, we deliberate on the value of PHR technologies to consumers by highlighting their applications across the spectrum of health care delivery activities. Finally, we provide a holistic summary of the value propositions and benefits of PHR systems to various health care industry constituents, including consumers, providers, payors, and public health agencies.

Our review indicates that PHR systems have made considerable progress over the past decade in terms of technology features and functions available at the product level. Compared with early PHR products that simply offer a basic functionality to maintain PHI [47,70], contemporary technologies offer a myriad of content-, connectivity-, and collaboration-based features and functions. Consequently, in recent years, the academic community has paid increasing attention to PHR functionality related to health monitoring tools, social networking features, and CDS functions.

From a value perspective, our analysis demonstrates that the value-generating potential of PHR systems arises from their role as an enabler for the integration of health care delivery activities across the full cycle of care for the health care consumer. These technologies can offer a useful mechanism for information exchange and care coordination among providers, thus leading to improved health outcomes for consumers. The consumer value framework conceptualized in this paper highlights that PHR functions have the potential to enhance patient experience through various touchpoints in health care delivery.

Our review also shows that although *consumers* are the primary beneficiaries of the functionality provided by PHR technologies, their overall value and benefits span across the activities and constituents of the health care delivery chain. At the consumer level, PHR systems can facilitate improved consumer health outcomes through the self-management of health and wellness as well as through enhanced quality of care. Moreover, these technologies can also generate channel partner value for providers and payors by enabling operational efficiency and reducing the cost of care. Finally, long-term and effective use of PHRs can also produce societal value in the form of improved public health outcomes.

This study offers several opportunities for research and potential practical applications. In terms of future research directions, we encourage researchers to undertake an empirical assessment of our conceptualized functional utility and consumer value frameworks for PHR technologies. In particular, our literature review indicates a significant dearth of studies addressing the issue of consumer value of PHR offerings. Our study offers a possible starting point for this type of research. For health care practice, our review may be relevant to health care professionals associated with value analysis committees that are commissioned for the appraisal and recommendation of innovative HITs. A value framework such as the one proposed in this paper that integrates functional attributes, use cases, and applications in health care delivery activities can potentially be applied to the value assessment of other HITs as well.

## Appendix

Multimedia Appendix 1 Examples of personal health record use cases in the health care delivery value chain.

## Abbreviations

CDS: clinical decision support
CDVC: care delivery value chain
EHR: electronic health record
EMR: electronic medical record
HIS: health information system
HIT: health information technology
PHI: personal health information
PHR: personal health record
PMRI: patient medical record information
