# Supplementary material for: Utility, Value, and Benefits of Contemporary Personal Health Records: Integrative Review and Conceptual Synthesis
Source: J Med Internet Res. 2021 Apr 29;23(4):e26877. doi: 10.2196/26877 (PMC8120425; doi:10.2196/26877)
Supplement: Multimedia Appendix 1 [file jmir_v23i4e26877_app1.pdf]

# Appendix 1: Examples of Personal Health Record (PHR) Use-Cases in the Healthcare Delivery Value Chain

| PHR Features and Functions | Healthcare Delivery Value Chain Activities: Monitoring and Preventing | Diagnosing | Preparing | Intervening | Recovering and Rehabbing | Monitoring and Managing |
|---|---|---|---|---|---|---|
| **Personal Health Information Resources** | • Keeping patient diaries<br>• Referencing educational resources<br>• Recording self-exams | • Reviewing patient self-reported data | • Referencing educational resources | | • Keeping patient diaries<br>• Referencing educational resources<br>• Recording self-exams | • Keeping patient diaries<br>• Referencing educational resources<br>• Recording self-exams |
| **Patient and Provider Records** | • Reviewing medical history | • Reviewing medical history<br>• Updating PMRI<br>• Maintaining CCR | • Reviewing medical history | • Reviewing medical history<br>• Updating PMRI<br>• Maintaining CCR | • Updating PMRI<br>• Maintaining CCR | • Reviewing medical history<br>• Maintaining CCR |
| **Health Monitoring Tools** | • Tracking lifestyle health data<br>• Availing medical device data<br>• Responding to health alerts & notifications | • Responding to health alerts & notifications<br>• Availing medical device data | • Tracking lifestyle health data<br>• Availing medical device data | | • Tracking lifestyle health data<br>• Availing medical device data<br>• Responding to health alerts & notifications | • Tracking lifestyle health data<br>• Availing medical device data<br>• Responding to health alerts & notifications |
| **Contact and Communication Features** | • Availing reminders & appointments for regular checkups<br>• Connecting with specialists for early referrals | • Accessing diagnosis consultations & follow-ups<br>• Requisitioning lab tests<br>• Reporting remote monitoring data | • Consulting with designated clinicians | • Counselling from attending physicians<br>• Ensuring treatment compliance | • Counselling from attending clinicians<br>• Availing reminders & appointments for follow-ups<br>• Acquiring prescription refills<br>• Connecting with specialists for referrals<br>• Reporting remote monitoring data | • Availing reminders & appointments for follow-ups<br>• Acquiring prescription refills<br>• Reporting remote monitoring data |
| **Shared Access and Social Networks** | • Assessing family medical history<br>• Seeking information from online forums<br>• Receiving social support from online communities | • Obtaining second opinions online<br>• Assessing family medical history<br>• Contributing to crowdsourced illness data | • Receiving social support from online communities | | • Receiving social support from online communities | • Seeking information from online forums<br>• Contributing to crowdsourced illness data |
| **Decision Support Functions** | • Consulting with specialists<br>• Counselling from experts about future risks<br>• Availing early health warnings & CDS advisories | • Reviewing health risk profiles<br>• Recommending treatment alternatives<br>• Checking drug-drug or drug-allergy interactions | • Reviewing health risk profiles<br>• Checking drug-drug or drug-allergy interactions | • Developing tailored interventions | • Consulting with specialists<br>• Developing tailored interventions<br>• Resolving treatment side-effects | • Counselling from experts about long-term care<br>• Availing early health warnings & CDS advisories |
